# Short-Term Morphological Changes in Asymptomatic Perimandibular Muscles after Dry Needling Assessed with Rehabilitative Ultrasound Imaging: A Proof-of-Concept Study

**DOI:** 10.3390/jcm10020209

**Published:** 2021-01-08

**Authors:** Alice Botticchio, Firas Mourad, Samuel Fernández-Carnero, José Luis Arias-Buría, Alejandro Santodomingo Bueno, Juan Mesa Jiménez, Massimiliano Gobbo

**Affiliations:** 1 Poliambulatorio Physio Power, 25124 Brescia, Italy; firas.mourad@me.com; 2 Department of Clinical Science and Translational Medicine, University of Rome Tor Vergata, 00133 Roma, Italy; 3 Department of Physiotherapy, LUNEX International University of Health, Exercise and Sports, 4671 Differdange, Luxembourg; 4 Physical Therapy Department, Alcalá de Henares University, 28801 Alcalá de Henares, Spain; samuelfernandezcarnero@gmail.com; 5 Department of Physical Therapy, Occupational Therapy, Physical Medicine and Rehabilitation, Universidad Rey Juan Carlos, 28922 Alcorcón, Spain; joseluis.arias@urjc.es; 6 Clínica Physed S.Coop., 28051 Madrid, Spain; alejandro.santodomingo@gmail.com; 7 Faculty of Medicine, CEU San-Pablo University, 28668 Madrid, Spain; jmesaj@ceu.es; 8 Laboratory of Clinical Integrative Physiology, University of Brescia, 25123 Brescia, Italy; massimiliano.gobbo@unibs.it; 9 Department of Clinical and Experimental Sciences, University of Brescia, 25123 Brescia, Italy

**Keywords:** Rehabilitative Ultrasound Imaging, ultrasonography, dry needling, temporomandibular joint, temporomandibular joint disorders, masticatory muscles, Physical Therapy

## Abstract

Facial anatomical structures are not easily accessible to manual palpation. The aim of our study is to objectively assess temporomandibular joint and perimandibular muscles dimensions by means of sonographic measurements before and after dry needling (DN) in asymptomatic subjects. Seventeen subjects participated in this before-after study with a within-subject control. After random allocation, one side of the face was used for the intervention and the contralateral as control. DN was performed on the temporal, masseter, and sternocleidomastoid muscles. Each subject was examined bilaterally before, immediately after, and one month after the intervention through Rehabilitative Ultrasound Imaging (RUSI) of the temporomandibular articular disc and the three target muscles. Maximum mouth opening was measured at baseline and at one month. After a single DN session, articular disc thickness significantly decreased; muscles’ thicknesses (except for temporal thickness) significantly decreased immediately and at follow-up on the treated side; no significant changes resulted for the control side. The maximum mouth opening increased from 4.77 mm to 4.86 mm. RUSI may be useful to assess the dimensions and thickness of the temporomandibular disc and muscles before and after an intervention. DN influences muscle morphology, and it has a positive influence on mouth opening in the short term.

## 1. Introduction

Temporomandibular disorders (TMD) affects 5–12% of the general population and represent one of the most frequent causes of musculoskeletal pain and disability, second only to low back pain [1,2]. Orofacial pain and altered masticatory function are hallmarks of symptomatic TMD; individuals suffering from TMD commonly experience changes in multiple aspects of their biological and psychosocial functioning [3,4,5]. Among TMD, myofascial pain (single or multiple diagnoses) is the most frequent diagnosis (42% to 51.8%), followed by disc displacement with reduction (32.1%) or arthralgia (30%) [6].

Ultrasonography is a non-invasive diagnostic technique highly accurate and reliable in detecting cross-sectional changes of small muscles, which can be well-performed also by novice practitioners for these specific purposes [7,8]. Ultrasonography and electromyography studies have found a relationship between increased thickness and contraction activity of the masseter, temporal and sternocleidomastoid muscles in patients with TMD. Pain on the sternocleidomastoid muscle was significantly associated with myogenic TMD and increased electromyographic activity of this muscle [9,10]. Hypertrophy of the masseter and temporal muscles has been shown to alter the shape and thickness of these structures, leading to aesthetic changes of the face and to functional issues of the temporomandibular joint (TMJ). TMJ is the result of a multi-factorial condition (emotional stress, chronic bruxism, microtrauma, hyperfunction, and parafunction of the chewing muscles) and seems to be more common in certain ethnic subgroups [11]. Although hypertrophy of the chewing muscles represents a physiological finding, in some individuals, it seems to be associated with pain [12].

Recently, dry needling (DN) has been suggested as a promising intervention for the treatment of some facial structures not easily accessible to manual palpation [13]. The use of needles without injectate directed to masticatory muscles [14,15] relieves pain and tenderness in myofascial syndrome [16]. Turo et al., by adopting an index of mechanical heterogeneity applied to ultrasound imaging, observed a correlation between muscle tissue changes and the use of DN [17].

The aim of this proof-of-concept study is to objectively assess TMJ articular disk and short-term changes in the morphology of perimandibular muscles by means of sonographic measurements applied in rehabilitative practice (Rehabilitative Ultrasound Imaging, RUSI) before and after DN in asymptomatic subjects. We also investigated the reliability of RUSI performed by a novice and an expert practitioner. To the best of our knowledge, this is the first study to investigate the morphological changes of perimandibular muscles after a DN intervention. This preliminary study was conducted in view of future trials addressing the clinical application of RUSI and DN in patients diagnosed with TMD.

## 2. Experimental Section

This preliminary study with within-subject control was conducted following the Consolidated Standards of Reporting Trials [18]. The research protocol was approved as a preliminary study (no sample size calculation needed) by the Human Subjects Committee of the Universidad Francisco de Vitoria (01/2016) and was registered in clinicaltrials.gov https://www.clinicaltrials.gov/ (NCT04578626). The authors followed the principles outlined in the Declaration of Helsinki for this study [19].

### 2.1. Participants

In this proof-of-concept study, we included a convenience sample of subjects without a formal diagnosis of TMD, referring to no symptoms related to TMD. Thirty-three subjects were recruited from the Centro de Simulación Clinica Avanzada-Universidad Francisco de Vitoria, Madrid, Spain-between January and February 2020. Eligible subjects had to be asymptomatic in the face/head region at the time of the visit and in the previous six months. Patients were excluded in case of pregnancy; medical history of systemic disease; current pharmacological therapy; history of recurrent headache and/or neck pain; presence of orofacial pain or temporomandibular symptoms assessed with the Diagnostic Criteria for Temporomandibular Disorders (DC/TMD) [1]; or bruxism. All subjects provided informed consent before their participation in the study.

### 2.2. Measurements

Before any experimental procedures, all subjects completed a temporomandibular and neck habits questionnaire (i.e., chewing side preference and the most frequent lying and phone holding side) and underwent a physical examination to measure the maximum mouth opening (i.e., the distance between the inferior border of superior incisor teeth and the superior border of opposing inferior incisor teeth) by a surgical ruler. The sonographic evaluations were performed before (T0) and immediately after (T1) the intervention. After one month, a follow-up evaluation was conducted (T2). A comparison between a novice and an experienced physical therapist in ultrasonography was performed. To assess the possible effects of the intervention at a more functional level (even if DN was performed only on one side), we evaluated the maximum mouth opening (secondary outcome) at T0 and T2.

### 2.3. Randomization

After baseline examination, for each individual, the side to be treated was randomly assigned by using a computer-generated randomization process (software “epidat” version 3.1; DirecciónXeral de SaúdePública, Xunta de Galicia, Spain) prior to the beginning of the study. The contralateral side was used as control. The assessor and the subjects were masked to the allocation; however, based on the nature of the interventions, it was not possible to mask the treating physiotherapist. The statistician was informed about the side allocation only at the end of the data collection.

### 2.4. Ultrasonography Examination Procedures

**Figure 1:**
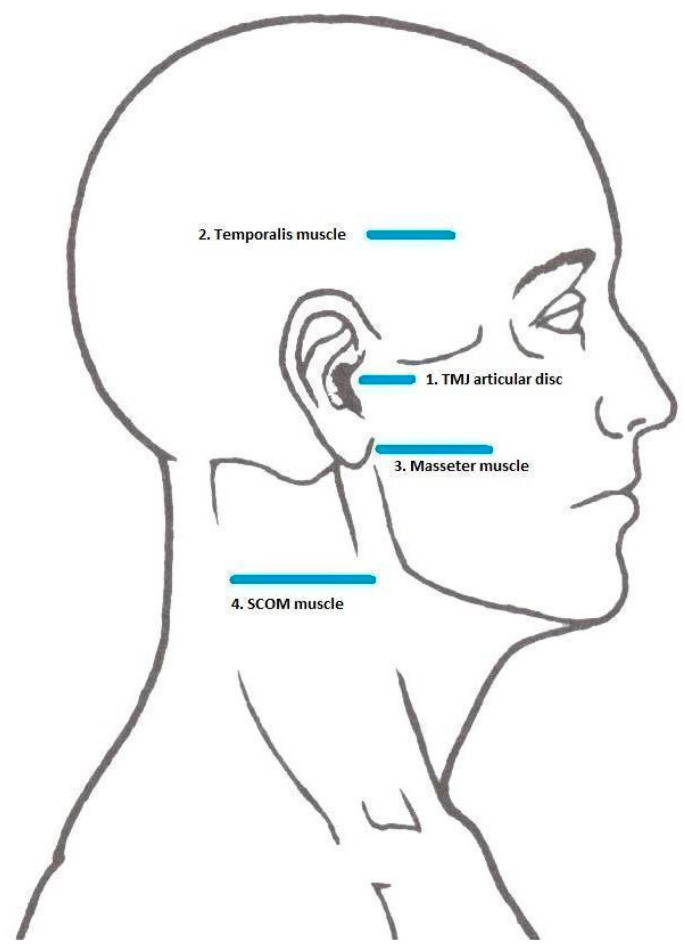
Reference Points for sonographic explorations.

All measurements were performed by an M9 Ultrasound System device (Mindray; Shenzhen, China) through a linear probe with a 40 mm footprint. Each individual was examined at T0, T1, and T2 on both sides of the face and the neck at four reference points (TMJ disc and temporal, masseter, sternocleidomastoid muscles) by the same masked clinician (Figure 1). The sonographic explorations were performed with the subjects lying in a supine position and the assessor sitting near the head of the subject. In line with previously published protocols, the transducer was held against the skin with light pressure anterior to the tragus and parallel to the von Camper’s line (the line running from the inferior border of the ala nasi to the superior border of the tragus) [20,21]. In order to correctly identify the target structures, patients were asked to: open and close the mouth for the TMJ disc assessment, to clench the jaw for the temporal and masseter muscles assessment; and to contralaterally rotate the head for the sternocleidomastoid muscle evaluation. The morphological outcomes investigated by RUSI were: thickness, width, and cross-sectional area (CSA). Table 1 reports the specific sonographic outcomes measured for each mentioned anatomical structure, their measurement sites, and details of the examination procedures.

Post-process image analysis was performed using the software ImageJ (Laboratory for Optical and Computational Instrumentation (LOCI), University of Wisconsin–Madison, Madison, WI, USA) [24].

### 2.5. Dry Needling Intervention

After the baseline ultrasonography evaluation and before treating the randomly selected side, a physiotherapist with seven years of experience in DN procedures explored each target muscle by manual palpation. Although the participants never complained of TMJ symptoms, the physiotherapist was able to identify painful taut bands in the assessed muscles. Subsequently, the skin surface covering the most painful areas were disinfected, and a sterile acupuncture needle (Seirin, J-type; 40–0.25 mm) was inserted perpendicular to the skin plane. The needle was manipulated in order to elicit local twitch responses [25] and then left in situ for 10 s. After needle removal, ischemic pressure was applied to prevent any bleeding.

### 2.6. Statistical Analysis

Data were imported to IBM SPSS, version 21 (Armonk, NY, USA), for statistical analysis. For the descriptive analysis, mean and standard deviation were calculated for all the quantitative variables, while percentage values were used to describe the qualitative ones. A two-way repeated-measures analysis of variance (ANOVA) with intervention as the between-subjects variable and time as the within-subjects variable was used to determine the changes obtained in the different RUSI measurements. Statistical significance was set at *p* < 0.01. Inter-Class Correlation (ICC) and ultrasonography inter-operator reliability were evaluated using Cronbach’s alpha.

## 3. Results

A total of thirty-three subjects were initially recruited for the study. Seven subjects did not meet the inclusion criteria, three declined to participate, one dropped out for other reasons, while the remaining twenty-two volunteers were recruited for the study. Both face sides for each subject were considered for a total of forty-four face sides, which were randomly divided into treated sides and control sides. An additional five subjects dropped out for personal reasons before the treatment.

**Figure 2:**
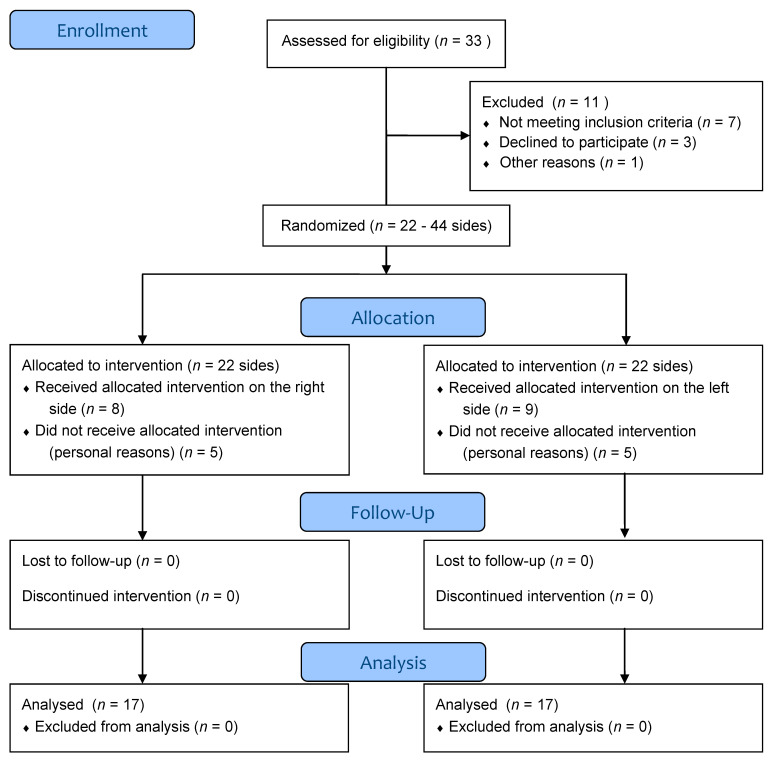
Flow Diagram.

**Figure 3:**
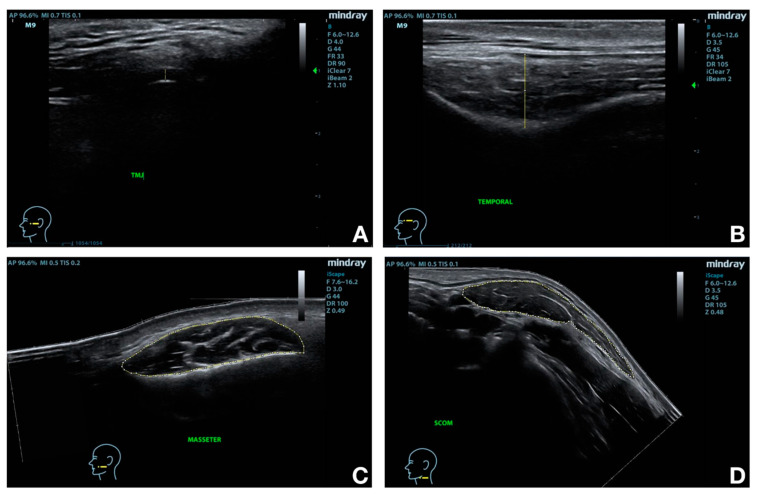
Target structures and main measurements. Temporomandibular joint (TMJ) articular disc thickness (**A**); Temporal muscle thickness (**B**); Masseter muscle cross-sectional area (CSA) (**C**); Sternocleidomastoid muscle CSA (**D**).

From the final seventeen participants, eight were treated on the right side and nine on the left side. All of them concluded the study and were included in the final data analysis (Figure 2). Baseline characteristics of the subjects are summarized in Table 2. Figure 3 shows sonographic images and applied measurements obtained from a representative subject.

### 3.1. Morphological Changes in the Intervention Side

DN intervention significantly influenced the outcomes (Table 3). TMJ disc thickness significantly decreased (*p* < 0.001) at T1, although it returned to pre-treatment values at T2. Temporal muscle thickness significantly decreased (*p* = 0.003) after the treatment and returned to baseline values at T2. The mean masseter and sternocleidomastoid thicknesses significantly decreased (*p* < 0.001; *p* = 0.005) immediately after DN, with a further reduction at T2. The mean masseter and sternocleidomastoid width did not significantly change at T1 and at T2. The mean masseter CSA significantly decreased (*p* < 0.001) at T1 and T2, while mean sternocleidomastoid CSA significantly decreased (*p* = 0.007) only at T2.

### 3.2. Morphological Changes in the Control Side

Table 4 reports the outcomes collected from the control side. No significant changes were found between T0, T1, and T2.

### 3.3. Inter-Class Correlation

The comparison between the measurements performed by the expert and the novel operators reported Cronbach’s alpha values higher than 0.70 for all the measured items (Table 5).

### 3.4. Functional TMJ Observation

The mean value of maximum mouth opening increased from 4.771 mm (standard deviation (SD) ± 0.436) at T0 to 4.859 mm (±0.412) at T2. The changes were not statistically significant (*p* = 0.261).

## 4. Discussion

To the best of our knowledge, this is the first study to investigate morphological changes in perimandibular muscles following DN intervention. Through the application of RUSI, we were able to detect the short-term changes of the temporomandibular disk and the target muscles’ morphology induced by a single session of DN. The treated muscles appeared significantly thinner with respect to baseline assessment. We explain these findings by assuming that muscle tone was reduced as a result of the DN intervention on the palpable taut bands within the muscle tissue. We further suggest that the resulting muscle changes influenced, in turn, the articular disk thickness observed after DN. Nevertheless, the disk thickness returned to baseline values after one month while the thickness and CSA of the temporomandibular muscles were consistently reduced at T2. It is difficult to compare our findings with previous studies as our work is the first study evaluating structural, morphological changes of the temporomandibular region following DN. Smith et al. found that DN was effective in increasing maximum mouth opening in patients suffering from TMD of myogenic origin [26]. Although not statistically significant, our findings observed an improvement in mouth’s opening despite the participants were not complaining of TMD, the intervention addressed only one side of the face, and one single DN session was provided.

Ultrasonography is a low cost and non-invasive imaging technique that has the potential to impact daily clinical practice by reducing the risk of radiation exposure typical of other imaging investigations [27]. Although ultrasonographic imaging is operator-dependent, we found that RUSI had good-to-excellent inter-observer reliability (Cronbach’s alpha values > 0.7, with many values higher than 0.9) in evaluating the cross-sectional area and thickness of the investigated muscles and the articular disc. Previously, other research teams demonstrated the efficacy of ultrasonography in evaluating the morphology of the hand and neck muscles [7,28]. Since 1995, Hides et al. suggested measuring the cross-sectional area of large muscles using panoramic view sonography, underling the advantages of this method compared to the more traditional measuring tape used by physiotherapists [27]. Teyhen et al. were the first to describe the impact of RUSI in physiotherapists’ clinical practice for monitoring tissue changes after manual physiotherapy interventions [29]. More recently, ultrasonography was described as an adjunct to guide needle insertion and trigger point detection in patients suffering from myofascial pain syndrome [30].

### Strengths and Limitations

Although ultrasonography imaging is operator-dependent, the adoption of standardized references and a rigid methodology allowed us to collect highly comparable consecutive images and heighten the reliability of our findings. Moreover, the good overlapping of novice and expert measurements strengthened our findings on RUSI reliability.

Our study has limitations. Although the procedure was highly accurate, for each studied anatomical structure, we performed single morphological measurements instead of three consecutive measurements, which may affect the level of precision in the evaluations. The short term follow-up does not allow to fully understand the persistent effects provided by DN. Also, only one intervention was administered to each participant, thus likely not showing the full potential of the technique. We suppose that the within-subject randomization performed to assign the intervention and control side of the face did not permit to fully observe the mouth’s functional improvements although the treatment on one side was able to affect the whole mouth opening at T2 even if not significantly from the statistical point of view.

## 5. Conclusions

This is the first study to observe structural modifications of perimandibular tissues after DN. Our study confirmed RUSI’s feasibility and reliability for assessing immediate morphological changes of the treated anatomical structures in rehabilitative settings for both expert and novice users. Although our proof-of-concept study observed preclinical results, we hope that our findings will inform future trials aimed at determining if morphological changes after DN may be associated with modifications of symptoms and functional outcomes in patients with TMD.

## Figures and Tables

**Table 1:** Ultrasonographic outcomes and measurement procedures.

Anatomical Structure	Outcome	Measurement Site/Procedure
Temporomandibular joint disc	Thickness	At the point between the glenoid fossa of the temporal bone and the mandibular condyle cortical surface, anteriorly to the tragus cartilage. Measured as the distance between the opposing surfaces of the disc.
Temporal muscle	Thickness	At the level of the temporal fossa, one centimeter laterally, and one above the lateral edge of the eye [9]. Measured as the distance between the opposing borders of the muscle, along the transverse axis of the muscle.
Masseter muscle	Thickness	At the proximal third of the distance between the ear cartilage and mandible angle, just below the inferior connection of ear to face [22]. Measured as the distance between the superficial and the deep border of the muscle.
	Width	By a panoramic view function, moving the probe posteriorly-to-anteriorly following the curvature of the muscle and creating an image with an angle lower than 20°. Measured as the distance between the opposing extremities of the muscle along the transverse axis of the muscle.
	CSA	At the proximal third of the distance between the ear cartilage and mandible angle, just below the inferior connection of ear to face [22]. Evaluated by drawing the outline of the muscle on the image.
Sternocleidomastoid muscle	Thickness	Before the carotid artery bifurcation [23].
	Width	By a panoramic view function, moving the probe anteriorly-to-posteriorly following the surface of the muscle and creating an image with an angle lower than 25°.
	CSA	Before the carotid artery bifurcation [23]. Evaluated by drawing the outline of the muscle on the image.

CSA: cross-sectional area.

**Table 2:** Subjects’ baseline characteristics.

Gender (Male/Female)	12 (70.58%)/5 (29.41%)
Age (years)	22.18 ± 1.91
Height (m)	1.75 ± 0.06
Weight (Kg)	74.88 ± 12.72
BMI (Body Mass Index)	24.26 ± 3.30
Side of mouth more used to chew	R 64.71%/L 35.29%
Side of body more used to lie down	R 64.71%/L 29.41%/=5.88%
Side more used for telephone	R 88.24%/L 11.76%

**Table 3:** Ultrasound (US) measurements for the intervention side.

	T0	T1	T2	*p*-Value T0–T1	*p*-Value T1–T2	*p*-Value T0–T2
TMJ thickness (cm)	0.17 ± 0.04	0.13 ± 0.03	0.17 ± 0.04	**<0.001**	**<0.001**	0.878
Temporal thickness (cm)	1.12 ± 0.20	0.99 ± 0.21	1.21 ± 0.24	**0.003**	**<0.001**	0.099
Masseter thickness (cm)	0.58 ± 0.07	0.47 ± 0.06	0.44 ± 0.06	**<0.001**	**0.002**	**<0.001**
Masseter width (cm)	2.59 ± 0.13	2.62 ± 0.14	2.64 ± 0.11	0.501	0.385	0.115
Masseter CSA (cm^2^)	3.80 ± 0.25	3.55 ± 0.21	3.43 ± 0.19	**<0.001**	**0.004**	**<0.001**
SCOM thickness (cm)	0.44 ± 0.07	0.41 ± 0.09	0.38 ± 0.08	**0.005**	**0.007**	**<0.001**
SCOM width (cm)	2.76 ± 0.24	2.74 ± 0.2	2.77 ± 0.24	0.487	0.336	0.211
SCOM CSA (cm^2^)	3.88 ± 0.54	3.69 ± 0.65	3.60 ± 0.64	0.057	**0.017**	**0.007**

The values are expressed as mean ± standard deviation (statistically significant values highlighted in bold). before intervention: T0; immediately after intervention: T1; one month follow-up evaluation: T2; SCOM: sternocleidomastoid.

**Table 4:** US measurements for the control side. The values are expressed as mean ± standard deviation.

	T0	T1	T2	*p*-Value T0–T1	*p*-Value T1–T2	*p*-Value T0–T2
TMJ thickness (cm)	0.16 ± 0.04	0.16 ± 0.04	0.16 ± 0.04	0.959	0.964	0.855
Temporal thickness (cm)	1.14 ± 0.19	1.13 ± 0.21	1.22 ± 0.19	0.995	0.063	0.092
Masseter thickness (cm)	0.54 ± 0.05	0.52 ± 0.05	0.52 ± 0.05	0.414	0.999	0.440
Masseter width (cm)	2.54 ± 0.23	2.55 ± 0.2	2.53 ± 0.18	0.582	0.412	0.672
Masseter CSA (cm^2^)	3.66 ± 0.24	3.65 ± 0.22	3.64 ± 0.19	0.973	0.917	0.810
SCOM thickness (cm)	0.45 ± 0.08	0.45 ± 0.08	0.44 ± 0.08	0.956	0.705	0.527
SCOM width (cm)	2.74 ± 0.18	2.75 ± 0.15	2.73 ± 0.16	0.409	0.162	0.174
SCOM CSA (cm^2^)	3.94 ± 0.64	3.95 ± 0.6	3.90 ± 0.62	0.762	0.067	0.338

**Table 5:** Inter-operator reliability data with Cronbach’s alpha values.

	Cronbach’s Alpha		Cronbach’s Alpha
TMJ disc thickness R	0.997	TMJ disc thickness L	0.984
Temporalis thickness R	0.868	Temporalis thickness L	0.887
Masseter thickness R	0.886	Masseter thickness L	0.899
Masseter width R	0.887	Masseter width L	0.926
Masseter CSA R	0.883	Masseter CSA L	0.854
SCOM thickness R	0.929	SCOM thickness L	0.92
SCOM width R	0.895	SCOM width L	0.855
SCOM CSA R	0.973	SCOM CSA L	0.87

TMJ: temporomandibular joint; SCOM: sternocleidomastoid; CSA: cross-sectional area; R: right; L: left.

## Data Availability

The data presented in this study are available on request from the corresponding author. The data are not publicly available due to privacy.
